# Manual Reduction of Strangulated Internal Hemorrhoids Using Sugar: A Case Report

**DOI:** 10.7759/cureus.55929

**Published:** 2024-03-11

**Authors:** Goshi Fujimoto

**Affiliations:** 1 Gastroenterological Surgery, Koga Community Hospital, Yaizu, JPN

**Keywords:** case report, hemorrhoidectomy, sugar, manual reduction, strangulated hemorrhoids

## Abstract

Emergency hemorrhoidectomy for strangulated internal hemorrhoids should be avoided when possible. Manual reductions can relieve pain and prevent the need for emergency surgery.

Herein, we present a 51-year-old female patient. Over the preceding 20 years, she experienced prolapsed internal hemorrhoids every time she defecated and had been performing manual reductions herself. Due to significant pain and difficulty during the manual reduction of the hemorrhoids, she was transported to the emergency room. Anal inspection revealed prolapsed internal hemorrhoids and partial congestion. After 10 minutes of applying Lidocaine Hydrochloride Jelly 2% and *Escherichia coli *culture suspension/hydrocortisone ointment, the manual reduction was still difficult. Based on previous reports of using sugar to reduce stomal prolapse, we applied sugar directly to the hemorrhoids. Ten minutes later, the number of prolapsed hemorrhoids decreased, and manual reduction was possible. After one day of hospitalization for bed rest, the patient was discharged once it was confirmed that there was no prolapse of the internal hemorrhoids and that her pain had improved. Two weeks later, a grade III internal hemorrhoid was observed, which had markedly reduced in size compared with the time of admission.

Using sugar to reduce strangulated internal hemorrhoids manually can be useful due to its simplicity, minimal invasiveness, and cost-effectiveness.

## Introduction

Emergency hemorrhoidectomy may be considered for acute or severe cases of strangulated internal hemorrhoids. However, emergency surgery is associated with earlier complications and a higher risk of reoperation and anal stenosis compared with elective surgery [1,2]. Manual reduction of the prolapsed hemorrhoids is important during emergency surgery to reduce edema [3]. We report a case of manual reduction of strangulated internal hemorrhoids using sugar, which may be useful as it is easy to perform, minimally invasive, and inexpensive.

## Case presentation

**Figure 1:**
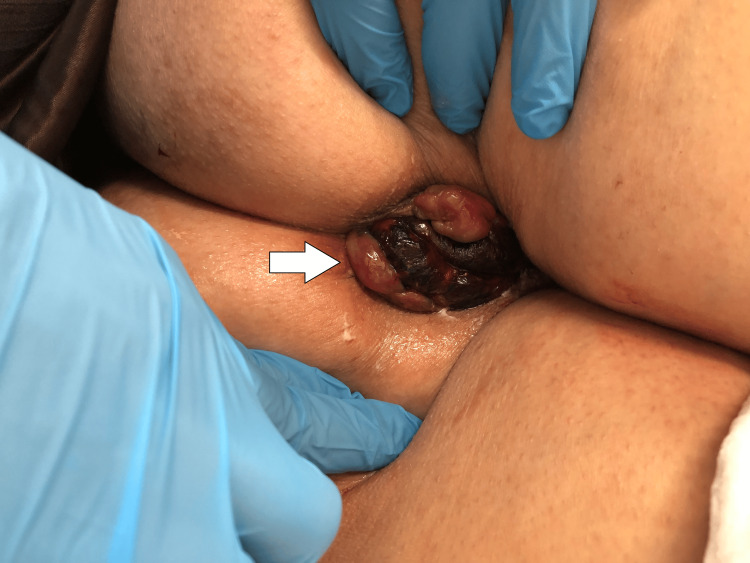
An anal inspection An anal inspection revealed a prolapsed internal hemorrhoids (arrow) and partial congestion

We report the case of a 51-year-old female patient with a medical history of obesity (body mass index of 30.0 kg/m2) and internal hemorrhoids who was transported to the emergency room because of difficulty and pain during the manual reduction of hemorrhoids. She experienced prolapsed internal hemorrhoids every time she defecated and had been performing manual reduction herself for 20 years. The patient’s condition did not improve at the time of admission. This was the first time that the patient had strangulated internal hemorrhoids and was unable to achieve manual reduction by herself. The prolapsed hemorrhoids had never become infected. Anal inspection revealed prolapsed internal hemorrhoids and partial congestion (Figure 1) without bleeding or discharge.

**Figure 2:**
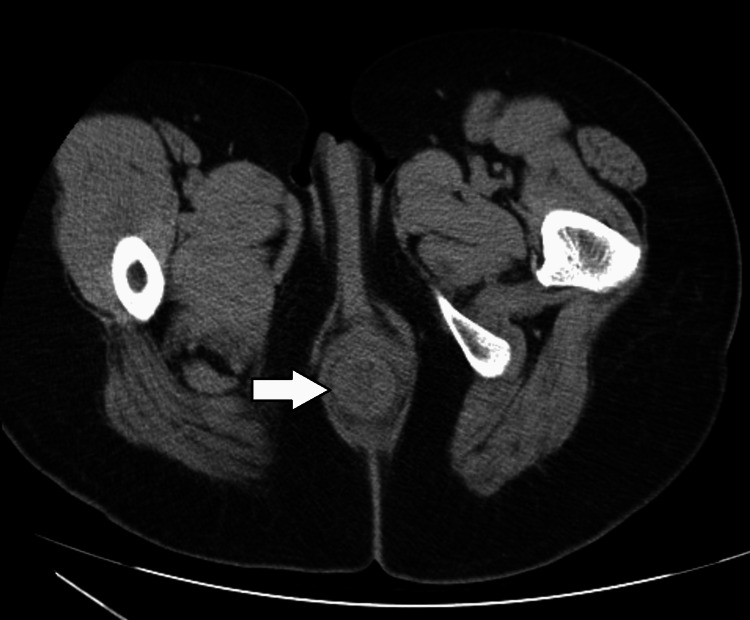
Computed tomography of the perianal region Computed tomography of the perianal region in the supine position revealed no obvious mass lesions in the rectum or anus, and rectal prolapse was suspected (arrow)

There were no significant findings on the perianal skin. A proctoscopy was not performed due to severe pain. Computed tomography of the perianal region revealed no obvious masses in the rectum or anus, and rectal prolapse was suspected (Figure 2).

**Figure 3:**
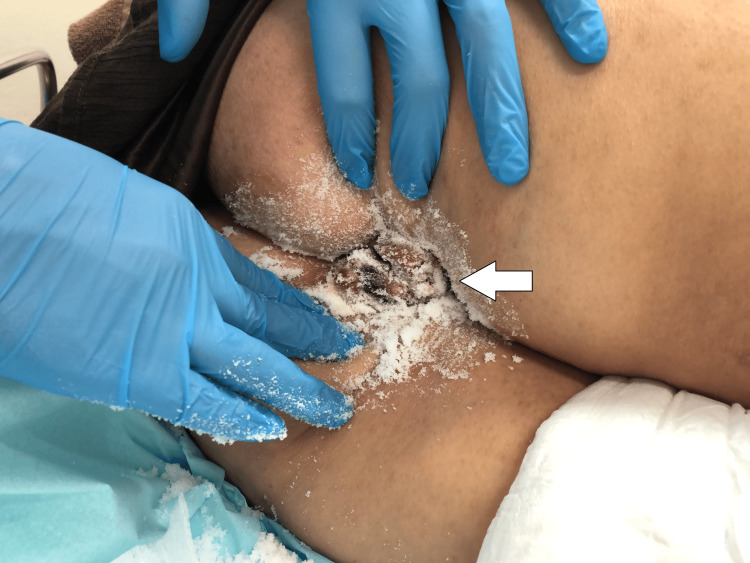
Application of sugar Application of fine-granulated ordinary table sugar directly on the hemorrhoids

Even after 10 minutes of applying Lidocaine Hydrochloride Jelly 2% and *Escherichia coli* culture suspension/hydrocortisone ointment, the manual reduction was difficult. Based on previous reports of using sugar to reduce stomal prolapse, we attempted to reduce the prolapsed hemorrhoids by pouring fine-granulated ordinary table sugar directly on the hemorrhoids (Figure 3).

**Figure 4:**
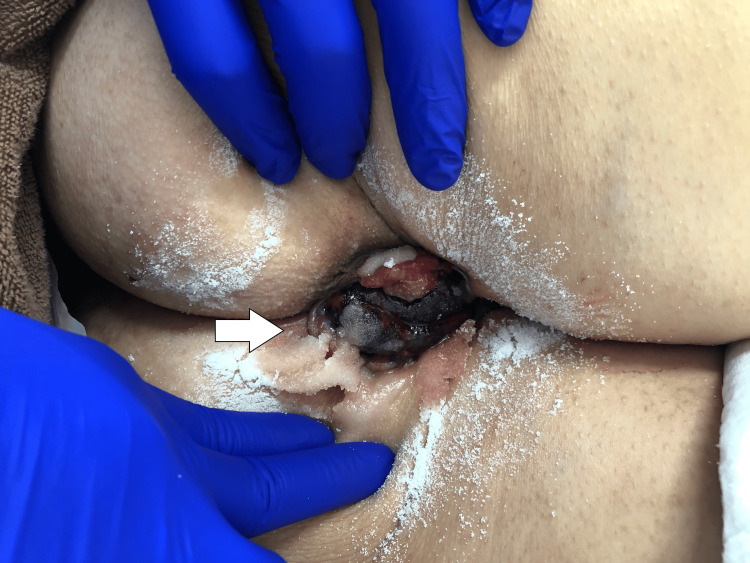
After the application of sugar The amount of prolapsed hemorrhoids decreased following the application of sugar

**Figure 5:**
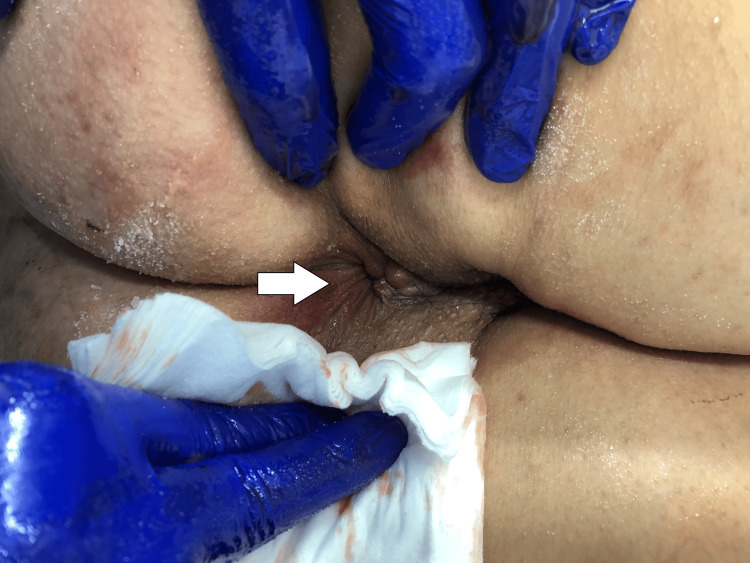
After complete manual reduction After complete manual reduction

Ten minutes later, the volume of prolapsed hemorrhoids had decreased, and manual reduction was possible (Figures 4-5).

After one day of hospitalization for bed rest, the patient was discharged after confirming that there was no further prolapse of internal hemorrhoids and that the pain had improved. An *Escherichia coli* culture suspension/hydrocortisone ointment was applied during the hospital stay, and after discharge, it was prescribed for one week, and the patient was instructed to rest in the lateral decubitus position. After two weeks, a painless grade III internal hemorrhoid was observed, which had markedly reduced in size since admission. The patient had an intense fear of surgery and had not visited the hospital in the past. We recommended surgery again; however, she did not wish to undergo surgery.

## Discussion

Compared with elective surgery, emergency hemorrhoidectomy for strangulated internal hemorrhoids involves earlier complications and a higher risk of reoperation and anal stenosis [1,2]. If manual reduction cannot be performed, an emergency hemorrhoidectomy is usually required. Unless tissue necrosis has occurred, the mucosa and anoderm should be preserved as much as possible to prevent postoperative anal stenosis [3,4]. Compression and manual reduction of prolapsed hemorrhoids are important during emergency surgery to reduce edema [4]. Conservative treatments include rest, analgesics, anti-inflammatory drugs, warm baths, topical applications, stool softeners, and antibiotics, which can help avoid emergency surgery. There are various therapeutic interventions for the internal anal sphincter during manual reduction, including relaxation and reduction of internal anal sphincter tone using sphincterotomy and exogenous nitric oxide, respectively; topical application of lidocaine chlorohydrate 2.5% gel; and limited resection of the largest hemorrhoid using four-finger anal stretching [2,5-7].

Sugar application has been shown to successfully reduce entrapped or prolapsed rectum, as well as colonic and small intestinal stomas, through its osmotic effect [8]. This follows the Stirling equation for fluid shifting from the hemorrhoid interstitium to outside of the hemorrhoid (Qfo):

Qfo = K [(Pi - Po ) - σ (πi - πo)]

where K is the filtration coefficient or leakiness of the mucous membrane to water, Pi is the hemorrhoid interstitial pressure, Po is the hydrostatic pressure outside the mucous membrane, σ is the protein reflection coefficient, πi is the interstitial protein osmotic pressure, and πo is the osmotic pressure outside the mucous membrane [9]. Here, sugar applied outside the mucosa increases πo, which increases Qfo and decreases hemorrhoidal prolapse. As Po decreases with water shifting, Qfo decreases, which lowers the hemorrhoidal volume reduction effect, representing the limitation of this therapy. This treatment has not caused side effects or allergies, except for a case of prolapsed hemorrhoid with persistent ischemic changes that required surgical management. Prolapsed rectums and internal hemorrhoids are covered by the mucus membrane; contrastingly, thrombosed external hemorrhoids are covered by the skin, which inhibits the water-shifting effect of sugar and thus impedes its efficacy [10]. Our case involved the manual reduction of strangulated internal hemorrhoids on the day of strangulation and was only a temporary crisis management measure. Further studies are warranted to confirm the efficacy and effectiveness of sugar for manual hemorrhoid reduction.

## Conclusions

Manual reduction using sugar for prolapsed rectum and stomas has been reported and was also effective for strangulated internal hemorrhoids in this case. Using sugar for strangulated internal hemorrhoids may facilitate manual reduction through its osmotic effect and potentially prevent the need for emergency surgeries. This intervention may be useful as it is easy to perform, minimally invasive, and inexpensive.
